# Hospital Frailty Risk Score predicts adverse events in revision total hip and knee arthroplasty

**DOI:** 10.1007/s00264-021-05038-w

**Published:** 2021-04-15

**Authors:** Matthias Meyer, Timo Schwarz, Tobias Renkawitz, Günther Maderbacher, Joachim Grifka, Markus Weber

**Affiliations:** 1 Department of Orthopedic Surgery, Regensburg University Hospital, Asklepios Klinikum Bad Abbach, Kaiser-Karl V.-Allee 3, 93077 Bad Abbach, Germany; 2 Heidelberg University Orthopedic Hospital, Heidelberg, Germany

**Keywords:** HFRS, Revision arthroplasty, Adverse events, Outcome, Frailty

## Abstract

**Introduction:**

The Hospital Frailty Risk Score (HFRS) is a validated risk stratification model referring to the cumulative deficits model of frailty. The purpose of this study was to evaluate the HFRS as a predictor of 90-day readmission and complications after revision total hip (rTHA) and knee (rTKA) arthroplasty.

**Methods:**

In a retrospective analysis of 565 patients who had undergone rTHA or rTKA between 2011 and 2019, the HFRS was calculated for each patient. Rates of adverse events were compared between patients with low and intermediate or high frailty risk. Multivariable logistic regression models were used to assess the relationship between the HFRS and post-operative adverse events.

**Results:**

Patients with intermediate or high frailty risk showed higher rates of readmission (30days: 23.8% vs. 9.9%, *p* = 0.006; 90days: 26.2% vs. 13.0%, *p* < 0.018), surgical complications (28.6% vs. 7.8%, *p* < 0.001), medical complications (11.9% vs. 1.0%, *p* < 0.001), other complications (28.6% vs. 2.3%, *p* < 0.001), Clavien-Dindo grade IV complications (14.3% vs. 4.8%, *p* = 0.009), and transfusion (33.3% vs. 6.1%, *p* < 0.001). Multivariable logistic regression analyses revealed a high HFRS as independent risk factor for surgical complications (OR = 3.45, 95% CI 1.45-8.18, *p* = 0.005), medical complications (OR = 7.29, 95% CI 1.72-30.97, *p* = 0.007), and other complications (OR = 14.15, 95% CI 5.16-38.77, *p* < 0.001).

**Conclusion:**

The HFRS predicts adverse events after rTHA and rTKA. As it derives from routinely collected data, the HFRS could be implemented automated in hospital information systems to facilitate identification of at-risk patients.

**Supplementary Information:**

The online version contains supplementary material available at 10.1007/s00264-021-05038-w.

## Introduction

Total hip arthroplasty (THA) and total knee arthroplasty (TKA) are safe and effective treatment options for patients with advanced osteoarthritis [1, 2]. Between 2007 and 2017, the frequency of THA and TKA increased by 30% and 40% respectively, in countries belonging to the Organization for Economic Co-operation and Development (OECD) [3]. Meanwhile, a trend to perform total joint arthroplasty in younger patients can be observed. By the year 2030, every second patient receiving THA or TKA is expected to be younger than 65 years, with the strongest increase projected for patients aged 45–55 years [4]. As implant failure rates and risk of revision inversely correlate with patient age [5], the number of revisions is expected to increase dramatically in the next decade [4, 6].

Revision total hip arthroplasty (rTHA) and revision total knee arthroplasty (rTKA) are cost-intensive surgical procedures [7] and entail a higher risk of adverse events than primary procedures [8]. As a consequence, thorough pre-operative risk stratification and optimization of modifiable risk factors is crucial to avoid additional burden to patients and the health care system. Regarding pre-operative risk stratification, the concept of frailty gains in importance [9]. A recently published review confirmed significant associations between frailty and poor outcome after primary THA and TKA [10].

The Hospital Frailty Risk Score (HFRS) is a validated geriatric comorbidity measure, deriving from routinely collected administrative data [11]. Referring to the cumulative deficits model of frailty [12], the HFRS is based on differently weighted codes of the 10th revision of the International Statistical Classification of Diseases and Related Health Problems (ICD-10) and showed excellent results in predicting adverse events after primary THA and TKA [13]. To the best of the authors’ knowledge, the HFRS has not yet been validated in the context of revision hip and knee arthroplasty.

Therefore, we aimed to evaluate the utility of the HFRS as a predictor of adverse events in a retrospective analysis of 565 rTHAs and rTKAs performed at a high-volume arthroplasty centre. We hypothesized that the HFRS correlates with readmission rate and post-operative complications.

## Methods

### Study design and study population

This is a retrospective study based on a database derived from the department’s joint registry and the hospital information system. The study was approved by the Ethics Committee of the Regensburg University Hospital, Regensburg, Germany (20-1821-104). From the database, all patients who had undergone rTHA and rTKA between January 2011 and December 2019 were included. Patients with incomplete data files were excluded.

Endpoints of the study were readmission within 30 and 90 days, complications, and transfusion. Follow-up for readmission in our department was 90 days. Complications were categorized in surgical (major bleeding with need for transfusion, periprosthetic fracture, wound healing disorder, wound infection, dislocation), medical (myocardial infarction, decompensated heart failure, cardiac arrhythmias, pneumonia, renal failure), and other complications (collapse, thrombosis, pulmonary embolism, delirium, cerebrovascular accident). Furthermore, complications were categorized according to the Clavien-Dindo-Classification [14]. This classification system ranks complications into five grades, based on the therapy used for correction. Any deviation from the normal post-operative course without the need for pharmacological treatment or surgical, endoscopic, and radiological intervention represents a grade I complication. Grade II complications require specific pharmacological treatment, whereas grade III complications result in surgical, endoscopic, or radiological intervention. Grade IV complications are defined as life-threatening events requiring intensive care management. Grade V represents the death of a patient [14].

### Surgical techniques

All operations were performed in a single Department of Orthopedic Surgery of a University Medical Center. Revision total hip arthroplasty was performed in the supine decubitus position using a lateral Hardinge approach. Revision total knee arthroplasty was performed through a medial parapatellar approach. Data of the components implanted for revision arthroplasty were not available in the database.

### Data collection

Principal and secondary diagnoses at the time of hospitalization were extracted from the hospital information system (ORBIS®; Agfa Healthcare) including corresponding ICD-10-Codes. Diagnostic codes had been entered by professional clinical coders and were double-checked by physicians using information gathered from patients’ medical records. Further available data from our clinical information system were age, gender, length of stay, transfusion, transfer to intensive care unit, reoperation, and readmission. Operative procedure and complications were extracted from the department’s joint registry.

### Calculation of the Hospital Frailty Risk Score

The Hospital Frailty Risk Score was calculated retrospectively for every included patient based on the available ICD-10-Codes that were entered for the time of admission. ICD-10-Codes from previous stays were also included. Unknown or undiagnosed comorbidities could not be coded and therefore did not contribute to the calculation of the HFRS. The Score derives from 109 ICD-10-Codes that were identified characteristic for a cluster of frail individuals [11]. Dependent on how strong each ICD-10-Code predicted membership in the cluster of frail patients, different points were awarded to each code and summed up to a maximum possible score of 173.2 points [11]. According to literature, patients were divided into the frailty risk categories low (HFRS below 5 points), intermediate (HFRS 5 to 15 points), and high (HFRS above 15 points) [11]. The ICD-10-Codes and weighting factors to derive the HFRS as recommended by Gilbert et al. are provided in the Supplementary information [11].

### Statistics

For statistical analysis, continuous data are presented as mean (standard deviation). Group comparisons were performed by two-sided *t*-tests. Absolute and relative frequencies were given for categorical data and compared between groups by chi-square tests. The primary hypothesis in the study was tested on 5% significance level. For all secondary hypotheses, significance levels were adjusted according to Bonferroni [15]. Multivariable logistic regression analyses were conducted to assess whether the HFRS is a significant predictor of surgical complications, medical complications, other complications, and Clavien-Dindo IV complications while controlling for other variables known to be associated with adverse surgical outcomes such as surgery site, operative time, gender, age, and ASA classification [16, 17]. IBM SPSS Statistics 25 (SPSS Inc., Chicago, IL, USA) was used for analysis.

## Results

**Fig. 1:**
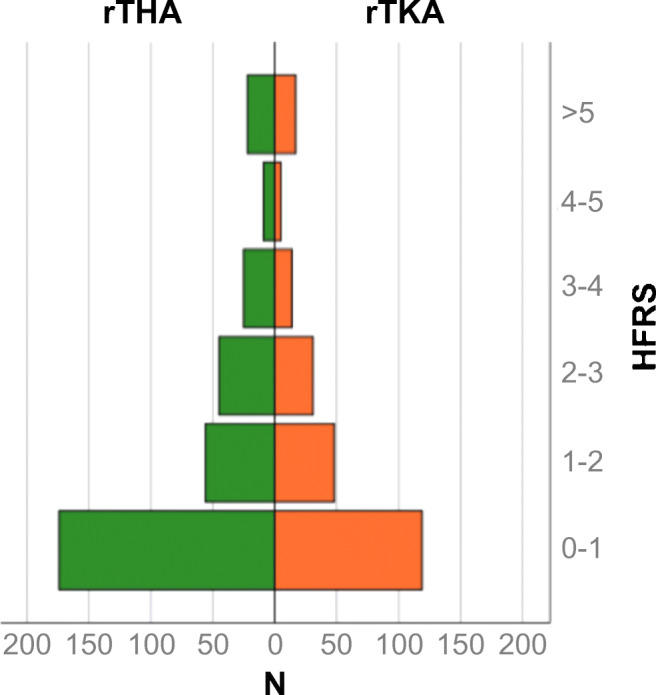
Distribution of the Hospital Frailty Risk Score (HFRS) in the revision total hip arthroplasty (rTHA) and revision total knee arthroplasty (rTKA) cohort

**Fig. 2:**
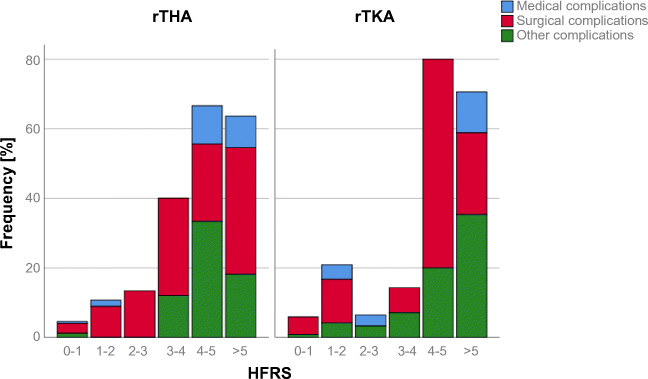
In patients undergoing revision total hip and knee arthroplasty, the frequency of readmission, transfusion, and Clavien-Dindo IV complications increased as Hospital Frailty Risk Score (HFRS) increased

**Fig. 3:**
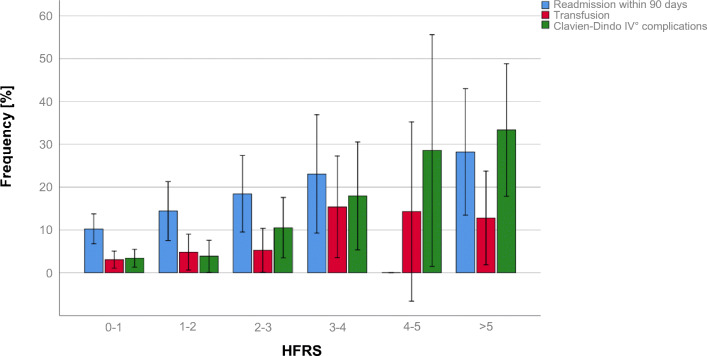
In patients undergoing rTHA or rTKA, the frequency of complication increased as HFRS increased

There were 331 and 234 patients who underwent rTHA and rTKA, respectively, during the study period. Demographic characteristics of the study group are shown in Table 1. Mean HFRS in the study group was 1.5 (1.5 ± 2.2). A total of 92.6% (523/565) of patients were categorized as low risk (HFRS <5), 7.3% (41/565) as intermediate risk (HFRS 5-15), and 0.2% (1/565) as high risk. HFRS distribution in the study group is shown in Fig. 1. Due to the limited number of high-risk patients in the study group, the intermediate and high-risk group were pooled for further analysis. Rates of measured adverse events increased with increasing HFRS in rTHA and rTKA (Figs. 2 and 3).

**Table 1:** Characteristics of the study group*

Demographics	rTHA	rTKA
*N*	331	234
Age (years)	68.7 ± 12.8	68.6 ± 9.6
Gender (female)	56.3%	58.8%
HFRS	1.5 ± 2.2	1.5 ± 2.3
ASA classification 1	10.9%	2.4%
ASA classification 2	45.2%	52.8%
ASA classification 3	43.2%	44.3%
ASA classification 4	0.7%	0.5%
Surgery duration (min)	140.2 ± 71.5	118.0 ± 57.2
Length of hospital stay (d)	14.0 ± 14.1	12.4 ± 8.2

*Values of categorical data are given as relative frequencies. Values of quantitative data are given as mean ± standard deviation

*rTHA* revision total hip arthroplasty. *rTKA* revision total knee arthroplasty, *HFRS* Hospital Frailty Risk Score, *ASA* American Society of Anesthesiologists

Readmission rate within 30 and 90 days after revision arthroplasty was 11.0% (62/565) and 14.0% (79/565), respectively. Compared to the low frailty risk group, the intermediate or high frailty risk group showed an absolute increase in readmission rate of 13.7% (23.8% [10/42] vs. 9.9% [32/523], *p* = 0.006) and 13.2% (26.2% [11/42] vs. 13.0% [68/523], *p* = 0.018) within 30 and 90 days, respectively.

The rates for surgical (absolute increase 20.8%, *p* < 0.001), medical (10.9%, *p* < 0.001), other (26.3%, *p* < 0.001), and Clavien-Dindo grade IV complications (9.5%, *p* = 0.009) were higher for patients with intermediate and high frailty risk, compared to patients with low frailty risk (Table 2). Furthermore, transfusion rate showed an absolute increase of 27.2% *r* in patients with intermediate or high frailty risk compared to patients with low frailty risk (*p* < 0.001; Table 2).

**Table 2:** Adverse events after revision total hip and knee arthroplasty according to frailty risk*

Adverse events	Low frailty risk	Intermediate or high frailty risk	*p-value*
Readmission within 30 days	9.9% (32/523)	23.8% (10/42)	0.006
Readmission within 90 days	13.0% (68/523)	26.2% (11/42)	0.018
Surgical complications	7.8% (41/523)	28.6% (12/42)	< 0.001
-Periprosthetic fracture	5.0% (26/523)	11.9% (5/42)	0.058
-Dislocation	1.3% (7/523)	7.1% (3/42)	0.006
-Wound healing disorder	2.1% (11/523)	7.1% (3/42)	0.043
-Joint infection	0.0% (0/523)	2.4% (1/42)	< 0.001
Medical complications	1.0% (5/523)	11.9% (5/42)	< 0.001
-Acute coronary syndrome	0.0% (0/523)	2.4% (1/42)	< 0.001
-Decompensated heart failure	0.2% (1/523)	4.8% (2/42)	< 0.001
-Cardiac arrhythmia	0.4% (2/523)	7.1% (3/42)	< 0.001
-Acute renal failure	0.4% (2/523)	2.4% (1/42)	0.086
Other complications	2.3% (12/523)	28.6% (12/42)	< 0.001
-Thrombosis	0.4% (2/523)	0.0% (0/42)	0.688
-Pulmonary embolism	0.4% (2/523)	0.0% (0/42)	0.688
-Collapse	0.6% (3/523)	2.4% (1/42)	0.179
-Cerebrovascular accident	0.6% (3/523)	0.0% (0/42)	0.623
-Postoperative delirium	0.4% (2/523)	26.2% (11/42)	< 0.001
Clavien-Dindo IV complications	4.8% (25/523)	14.3% (6/42)	0.009
Transfusion	6.1% (32/523)	33.3% (14/42)	< 0.001

*Values of categorical data are given as relative and absolute frequencies

The subgroup analyses of rTHA and rTKA also showed higher rates of adverse events in patients with intermediate or high frailty risk compared to patients with low frailty risk. However, the differences in readmission rate after rTHA as well as the differences in rates of Clavien-Dindo grade IV complications and readmission after rTKA did not reach statistical significance (Tables A.1, A.2 in the Supplementary information).

### Multivariable logistic regression analysis

Multivariable logistic regression analysis showed that HFRS was independently associated with surgical (OR 3.45, 95% CI 1.45–8.18, *p* = 0.005), medical (OR 7.29, 95% CI 1.72–30.97, *p* = 0.007), and other complications (OR 14.15, 95% CI 5.16–38.77, *p* < 0.001) (Table 3). Only Clavien-Dindo IV complications were not independently associated with HFRS (OR 2.34, 95% CI 0.78–6.96, *p* = 0.128).

**Table 3:** Multivariable analysis of risk factors associated with surgical, medical, other, and Clavien-Dindo IV complications after revision total hip and knee arthroplasty

Variable	OR	95% CI		*p*-value
Surgical complications				
Knee arthroplasty	1.156	0.605	2.210	0.661
Operative time	1.006	1.002	1.011	0.005
Gender (male)	0.693	0.358	1.341	0.276
Age	0.997	0.970	1.026	0.858
ASA class	1.083	0.640	1.832	0.767
HFRS	3.445	1.451	8.178	0.005
Medical complications				
Knee arthroplasty	1.851	0.441	7.769	0.400
Operative time	1.001	0.990	1.013	0.809
Gender (male)	0.503	0.098	2.594	0.412
Age	1.025	0.955	1.101	0.492
ASA classification	3.057	0.720	12.974	0.130
HFRS	7.287	1.715	30.968	0.007
Other complications				
Knee arthroplasty	1.677	0.660	4.263	0.277
Operative time	0.998	0.990	1.006	0.663
Gender (male)	1.891	0.722	4.952	0.194
Age	1.062	1.008	1.120	0.025
ASA class	0.621	0.287	1.341	0.225
HFRS	14.146	5.162	38.770	< 0.001
Clavien-Dindo IV complications				
Knee arthroplasty	0.798	0.336	1.894	0.609
Operative time	1.002	0.996	1.008	0.455
Gender (male)	1.187	0.516	2.731	0.686
Age	1.037	0.992	1.085	0.109
ASA class	1.938	0.919	4.088	0.082
HFRS	2.399	0.801	7.181	0.118

*OR* odds ratio, *CI* confidence interval, *preop* preoperative, *ASA* American Society of Anesthesiologists, *HFRS* Hospital Frailty Risk Score

## Discussion

Due to rising numbers of primary THA and TKA especially in younger patients [3, 4], a dramatic increase of patients in need for revision arthroplasty is projected for the next decades [4, 6]. Revision total hip and knee arthroplasty are cost-intensive and risky surgical procedures [7, 8]. Pre-operative risk stratification seems crucial to avoid additional burden to patients and health care providers. The Hospital Frailty Risk Score is a promising geriatric risk stratification model deriving from routinely collected administrative data [11] and showed excellent results in predicting adverse events after primary THA and TKA [13]. The aim of this study was to validate the HFRS with regard to adverse events after revision total hip and knee arthroplasty. We hypothesized that patients with intermediate or high frailty risk have higher rates of adverse events than patients with low frailty risk.

In this study, readmission rate within 90 days after surgery for patients with intermediate or high frailty risk was twice as high as for patients with low frailty risk. The rates for surgical complications, medical complications, other complications, Clavien-Dindo IV complications, and transfusion were three to 14 times higher in the cohort with intermediate or high frailty risk compared to the cohort with low frailty risk. Multivariable logistic regression analyses showed an independent association of the HFRS with all captured complications, except Clavien-Dindo IV complications. The findings of this study indicate that the HFRS is suitable to predict post-operative adverse events after revision total hip (rTHA) and knee (rTKA) arthroplasty.

Gilbert et al. developed the HFRS in order to provide hospitals with a geriatric assessment tool derived from routinely collected administrative data [11]. In the original validation cohort, consisting of over 1 million people aged over 75 and admitted to an acute hospital as an emergency, the HFRS performed excellent in predicting length of stay, readmission, and 30-day mortality [11]. To the best of the authors’ knowledge, this is the first study to evaluate the HFRS in the context of revision hip and knee arthroplasty. However, the HFRS has already been validated in primary total hip and knee arthroplasty. In a former study conducted by the authors, the HFRS was a strong predictor for reoperation within 90 days, readmission within 90 days, and post-operative complications after primary THA and TKA [13]. The current study group showed a mean HFRS of 1.5 and a mean age of 69 years, compared to 0.9 and 66 years, respectively, in the primary THA and TKA study group. These findings underline the importance of risk stratification in the revision arthroplasty as affected patients are older and suffer from more comorbidities.

Interestingly, multivariable logistic regression analysis revealed no independent association between the HFRS and Clavien-Dindo IV complications. A recent analysis of primary THA and TKA did not show independent associations between the HFRS and complications requiring ICU management either [13]. Moreover, in a retrospective analysis of 4381 patients admitted to ICU, Bruno et al. also found no association between the HFRS and adverse outcome [18]. As potentially life-threatening conditions, such as heart failure, renal failure, or chronic obstructive pulmonary disease, are weighted comparably low, the HFRS seems to be less suitable in predicting ICU admissions [13]. However, as multivariable regression analysis showed an OR of 2.34, an independent association between Clavien-Dindo IV complications and the HFRS could be present with higher number of cases.

Multiple other risk stratification models such as the Charlson Comorbidity Index (CCI), the Elixhauser Comorbidity Method (ECM), and the 5-Factor modified Frailty Index (mFI-5) have been validated in total hip and knee arthroplasty [17, 19–22]. In contrast, data regarding risk stratification in the context of revision hip and knee arthroplasty is limited. In a retrospective analysis of 13,948 and 16,304 patients who underwent rTHA and rTKA, respectively, Traven et al. found that the mFI-5 predicts serious medical complications, increased length of stay, discharge to a facility context, hospital readmission, and mortality [23]. Similar to the HFRS, the mFI-5 also corresponds to the cumulative deficits model of frailty [12]. Originally deriving from Rockwood’s Frailty Index [24], the mFI-5 has been condensed to 5 variables (diabetes, hypertension, congestive heart failure, chronic obstructive pulmonary disease, and dependent functional status) [25]. By contrast, the HFRS consists of 109 differently weighted variables. A recently published comparative study showed that the HFRS outperformed the mFI-5, as well as other current risk stratification models, in prediction of adverse events after primary THA and TKA [26]. It is assumed that the HFRS’s high number of differently weighted variables is responsible for its superior discriminative ability [13]. Although application of simple risk assessment tools seems more feasible in clinical practice, the HFRS holds the chance to be calculated automatically as it derives from routinely collected administrative data. The growing adoption of electronic health record systems might further simplify the application of sophisticated risk stratification models such as the HFRS in clinical practice [27]. The HFRS could be implemented automated in hospital information systems enabling identification of at-risk patients without extra effort or expense. Patients with high HFRS could be screened for modifiable risk factors, such as malnutrition or inadequately controlled diabetes mellitus. In a recently published retrospective analysis of 599 geriatric patients undergoing elective orthopedic surgery, 11% were malnourished and even 15% suffered from diabetes mellitus [28]. In a another retrospective analysis, Kee at al. found that 40% and 44% of patients who underwent total hip or knee arthroplasty, respectively, had at least one modifiable risk factor [29]. As part of an intensive surgery preparation, modifiable risk factors could be adjusted in order to minimize the perioperative risk of these vulnerable patients [30].

Compared to primary THA and TKA, revision total hip and knee arthroplasty entail a higher risk of adverse events [8]. In a recently published retrospective analysis of 8250 patients who had undergone primary THA or TKA in the authors’ department, rates of 90-day readmission, surgical complications, and Clavien-Dindo IV complications were 4.5%, 2.2%, and 1.6%, respectively [13]. Consequently, rates of adverse events were three to nine times higher in patients who underwent revision total hip or knee arthroplasty. These results are supported by Bohl et al., who found three to seven fold increased risks of systemic sepsis, deep wound infection, and organ infection in patients who had undergone rTHA and rTKA compared to primary THA and TKA [8]. The increased frequency of adverse events, extended surgery durations, prolonged length of stay, and higher implant costs result in high procedural charges for rTHA and rTKA. In a retrospective matched pair analysis Weber et al. found 76% higher costs for revision arthroplasty compared to primary procedures leading only to 24% higher reimbursement [7]. This underlines again the importance of pre-operative risk stratification and optimization of modifiable risk factors in revision arthroplasty. By use of risk stratification models such as the HFRS, orthopaedic surgeons are able to identify at-risk patients in an early stage of the treatment process. This holds the chance to adjust treatment planning and optimally prepare patients for surgery in order to minimize the risk of adverse events and avoid additional charges to the health care system.

This study has several limitations. Revision total hip and knee arthroplasty are very heterogeneous procedures. For example, the reason for revision varies from aseptic loosening over deep joint infection to periprosthetic fracture. Stratification of patients by the reason for revision surgery was not possible. Furthermore, diverse types of implants are used in revision arthroplasty and post-operative treatment protocols differ depending on the individual situation. These confounding factors represent a potential source of error. In addition, there are the usual limitations of a database study. Data acquisition was limited to the data available from the hospital information system and the institutional joint registry. Follow-up for readmission in our department was limited to 90 days after surgery. Readmissions to other hospitals could not be captured. Surgical complications could only be captured for 30 days post-operatively. Medical, other, and Clavien-Dindo IV complications could only be captured during hospital stay (mean 13 days). As the HFRS derives from ICD-10-Codes, over-coding and under-coding represent potential sources of bias. Furthermore, other parameters with possible influence on outcome, such as BMI and psychosocial aspects, could not be captured. Another limitation is the mean length of stay in our cohort. Considering international standards, the comparatively long length of stay is related to the German health care system and represents the typical length of stay after rTHA and rTKA during the study period. Despite these limitations, this study is the first to demonstrate the predictive ability of the HFRS in patients undergoing revision total hip and knee arthroplasty.

## Conclusion

The HFRS predicts adverse events like readmission and complications after revision total hip and knee arthroplasty. Patients undergoing revision arthroplasty are older, sicker, and more prone to complications than patients undergoing primary procedures. As the HFRS derives from routinely collected administrative data, it could be implemented automated in hospital information systems. Identification of at-risk patients at an early stage of the therapy process would hold the chance to optimize patient preparation for surgery in order to minimize the risk of adverse events and avoid additional charges to the health care system.

## Supplementary information

ESM 1 (DOCX 34 kb)

## Data Availability

The data that support the findings of this study are available from the corresponding author upon reasonable request.
