# Supplementary material for: Hospital Frailty Risk Score predicts adverse events in revision total hip and knee arthroplasty
Source: Int Orthop. 2021 Apr 15;45(11):2765–72. doi: 10.1007/s00264-021-05038-w (PMC8560670; doi:10.1007/s00264-021-05038-w)
Supplement: Supplementary file 1 — (DOCX 34 kb) [file 264_2021_5038_MOESM1_ESM.docx]

**Appendix**

**Appendix 1:** List of the 109 ICD-10 Codes used to calculate the Hospital Frailty Risk Score with points awarded for each Code as proposed by Gilbert et al [16]

| ICDCode | ICD Description | Points  awarded |
| --- | --- | --- |
| F00 | Dementia in Alzheimer's disease | 7.1 |
| G81 | Hemiplegia | 4.4 |
| G30 | Alzheimer's disease | 4.0 |
| I69 | Sequelae of cerebrovascular disease | 3.7 |
| R29 | Other symptoms and signs involving the nervous and musculoskeletal systems | 3.6 |
| N39 | Other disorders of urinary system | 3.2 |
| F05 | Delirium, not induced by alcohol and other psychoactive substances | 3.2 |
| W19 | Unspecified fall | 3.2 |
| S00 | Superficial injury of head | 3.2 |
| R31 | Unspecified haematuria | 3.0 |
| B96 | Other bacterial agents as the cause of diseases classified to other chapters | 2.9 |
| R41 | Other symptoms and signs involving cognitive functions and awareness | 2.7 |
| R26 | Abnormalities of gait and mobility | 2.6 |
| I67 | Other cerebrovascular diseases | 2.6 |
| R56 | Convulsions, not elsewhere classified | 2.6 |
| R40 | Somnolence, stupor and coma | 2.5 |
| T83 | Complications of genitourinary prosthetic devices, implants and grafts | 2.4 |
| S06 | Intracranial injury | 2.4 |
| S42 | Fracture of shoulder and upper arm | 2.3 |
| E87 | Other disorders of fluid, electrolyte and acid-base balance | 2.3 |
| M25 | Other joint disorders, not elsewhere classified | 2.3 |
| E86 | Volume depletion | 2.3 |
| R54 | Senility | 2.2 |
| Z50 | Care involving use of rehabilitation procedures | 2.1 |
| F03 | Unspecified dementia | 2.1 |
| W18 | Other fall on same level | 2.1 |
| Z75 | Problems related to medical facilities and other health care | 2.0 |
| F01 | Vascular dementia | 2.0 |
| S80 | Superficial injury of lower leg | 2.0 |
| L03 | Cellulitis | 2.0 |
| H54 | Blindness and low vision | 1.9 |
| E53 | Deficiency of other B group vitamins | 1.9 |
| Z60 | Problems related to social environment | 1.8 |
| G20 | Parkinson's disease | 1.8 |
| R55 | Syncope and collapse | 1.8 |
| S22 | Fracture of rib(s), sternum and thoracic spine | 1.8 |
| K59 | Other functional intestinal disorders | 1.8 |
| N17 | Acute renal failure | 1.8 |
| L89 | Decubitus ulcer | 1.7 |
| Z22 | Carrier of infectious disease | 1.7 |
| B95 | Streptococcus and staphylococcus as the cause of diseases classified to other chapters | 1.7 |
| L97 | Ulcer of lower limb, not elsewhere classified | 1.6 |
| R44 | Other symptoms and signs involving general sensations and perceptions | 1.6 |
| K26 | Duodenal ulcer | 1.6 |
| I95 | Hypotension | 1.6 |
| N19 | Unspecified renal failure | 1.6 |
| A41 | Other septicaemia | 1.6 |
| Z87 | Personal history of other diseases and conditions | 1.5 |
| J96 | Respiratory failure, not elsewhere classified | 1.5 |
| X59 | Exposure to unspecified factor | 1.5 |
| M19 | Other arthrosis | 1.5 |
| G40 | Epilepsy | 1.5 |
| M81 | Osteoporosis without pathological fracture | 1.4 |
| S72 | Fracture of femur | 1.4 |
| S32 | Fracture of lumbar spine and pelvis | 1.4 |
| E16 | Other disorders of pancreatic internal secretion | 1.4 |
| R94 | Abnormal results of function studies | 1.4 |
| N18 | Chronic renal failure | 1.4 |
| R33 | Retention of urine | 1.3 |
| R69 | Unknown and unspecified causes of morbidity | 1.3 |
| N28 | Other disorders of kidney and ureter, not elsewhere classified | 1.3 |
| R32 | Unspecified urinary incontinence | 1.2 |
| G31 | Other degenerative diseases of nervous system, not elsewhere classified | 1.2 |
| Y95 | Nosocomial condition | 1.2 |
| S09 | Other and unspecified injuries of head | 1.2 |
| R45 | Symptoms and signs involving emotional state | 1.2 |
| G45 | Transient cerebral ischaemic attacks and related syndromes | 1.2 |
| Z74 | Problems related to care-provider dependency | 1.1 |
| M79 | Other soft tissue disorders, not elsewhere classified | 1.1 |
| W06 | Fall involving bed | 1.1 |
| S01 | Open wound of head | 1.1 |
| A04 | Other bacterial intestinal infections | 1.1 |
| A09 | Diarrhoea and gastroenteritis of presumed infectious origin | 1.1 |
| J18 | Pneumonia, organism unspecified | 1.1 |
| J69 | Pneumonitis due to solids and liquids | 1.0 |
| R47 | Speech disturbances, not elsewhere classified | 1.0 |
| E55 | Vitamin D deficiency | 1.0 |
| Z93 | Artificial opening status | 1.0 |
| R02 | Gangrene, not elsewhere classified | 1.0 |
| R63 | Symptoms and signs concerning food and fluid intake | 0.9 |
| H91 | Other hearing loss | 0.9 |
| W10 | Fall on and from stairs and steps | 0.9 |
| W01 | Fall on same level from slipping, tripping and stumbling | 0.9 |
| E05 | Thyrotoxicosis [hyperthyroidism] | 0.9 |
| M41 | Scoliosis | 0.9 |
| R13 | Dysphagia | 0.8 |
| Z99 | Dependence on enabling machines and devices | 0.8 |
| U80 | Agent resistant to penicillin and related antibiotics | 0.8 |
| M80 | Osteoporosis with pathological fracture | 0.8 |
| K92 | Other diseases of digestive system | 0.8 |
| I63 | Cerebral Infarction | 0.8 |
| N20 | Calculus of kidney and ureter | 0.7 |
| F10 | Mental and behavioural disorders due to use of alcohol | 0.7 |
| Y84 | Other medical procedures as the cause of abnormal reaction of the patient | 0.7 |
| R00 | Abnormalities of heart beat | 0.7 |
| J22 | Unspecified acute lower respiratory infection | 0.7 |
| Z73 | Problems related to life-management difficulty | 0.6 |
| R79 | Other abnormal findings of blood chemistry | 0.6 |
| Z91 | Personal history of risk-factors, not elsewhere classified | 0.5 |
| S51 | Open wound of forearm | 0.5 |
| F32 | Depressive episode | 0.5 |
| M48 | Spinal stenosis | 0.5 |
| E83 | Disorders of mineral metabolism | 0.4 |
| M15 | Polyarthrosis | 0.4 |
| D64 | Other anaemias | 0.4 |
| L08 | Other local infections of skin and subcutaneous tissue | 0.4 |
| R11 | Nausea and vomiting | 0.3 |
| K52 | Other noninfective gastroenteritis and colitis | 0.3 |
| R50 | Fever of unknown origin | 0.1 |

**Appendix tables**

**Table A.1**: Adverse events after revision total hip arthroplasty according to frailty risk*

| Adverse Events | Low  frailty risk | Intermediate or high frailty risk | p-value |
| --- | --- | --- | --- |
| **Readmission within 30 days** | **12.4 % (38/307)** | **25.0 % (6/24)** | **0.079** |
| **Readmission within 90 days** | **15.3 % (47/307)** | **29.6 % (7/24)** | **0.077** |
| **Surgical complications**  - Periprosthetic fracture - Dislocation - Wound healing disorder | **8.1 % (25/307)**  5.9 % (18/307) 2.3 % (7/307) 1.0 % (3/307) | **33.3 % (8/24)**  8.3 % (2/24) 12.5 % (3/24) 12.5 % (3/24) | **< 0.001**  0.625 0.005 < 0.001 |
| **Medical complications**  - Acute coronary syndrome - Decompensated heart failure - Cardiac arrhythmia - Acute renal failure | **0.7 % (2/307)**  0.0 % (0/307) 0.3 % (1/307) 0.0 % (0/307) 0.3 % (1/307) | **12.5 % (3/24)**  4.2 % (1/24) 4.2 % (1/24) 8.3 % (2/24) 4.2 % (1/24) | **< 0.001**  < 0.001 0.019 < 0.001 0.019 |
| **Other complications**  - Pulmonary embolism - Collapse - Cerebrovascular accident - Postoperative delirium | **2.0 % (6/307)**  0.3 % (1/307) 0.3 % (1/307) 0.7 % (2/307) 0.7 % (2/307) | **25.0 % (6/24)**  0.0 % (0/24) 0.0 % (0/24) 0.0 % (0/24) 25.0 % (6/24) | **< 0.001**  0.779 0.779 0.692 < 0.001 |
| **Clavien Dindo IV° complications** | **5.5 % (17/307)** | **20.8 % (5/24)** | **0.004** |
| **Transfusion** | **8.5 % (26/307)** | **41.7 % (10/24)** | **< 0.001** |

* Values of categorical data are given as relative and absolute frequencies.

**Table A.2**: Adverse events after revision knee arthroplasty according to frailty risk*

| Adverse Events | Low  frailty risk | Intermediate or high frailty risk | p-value |
| --- | --- | --- | --- |
| **Readmission within 30 days** | **6.5 % (14/216)** | **22.2 % (4/18)** | **0.016** |
| **Readmission within 90 days** | **9.7 % (21/216)** | **22.2 % (4/18)** | **0.099** |
| **Surgical complications**  - Periprosthetic fracture - Wound healing disorder - Joint infection | **7.4 % (16/216)**  3.7 % (8/216) 3.7 % (8/216) 0.0 % (0/216) | **22.2 % (4/18)**  16.7 % (3/18) 0.0 % (0/18) 5.6 % (1/18) | **0.031**  0.013 0.406 0.001 |
| **Medical complications**  - Decompensated heart failure - Cardiac arrhythmia - Acute renal failure | **1.4 % (3/216)**  0.0 % (0/216) 0.9 % (2/216) 0.5 % (1/216) | **11.1 % (2/18)**  5.6 % (1/18) 5.6 % (1/18) 0.0 % (0/18) | **0.006**  0.001 0.093 0.772 |
| **Other complications**  - Thrombosis - Pulmonary embolism - Collapse - Cerebrovascular accident - Postoperative delirium | **2.8 % (6/216)**  0.9 % (2/216) 0.5 % (1/216) 0.9 % (2/216) 0.5 % (1/216) 0.0 % (0/216) | **33.3 % (6/18)**  0.0 % (0/18) 0.0 % (0/18) 5.6 % (1/18) 0.0 % (0/18) 27,8 % (5/18) | **< 0.001**  0.682 0.772 0.093 0.772 < 0.001 |
| **Clavien Dindo IV° complications** | **3.7 % (8/216)** | **5.6 % (1/18)** | **0.695** |
| **Transfusion** | **2.8 % (6/216)** | **22.2 % (4/18)** | **< 0.001** |

* Values of categorical data are given as relative and absolute frequencies.
